# The Effect of Increasing Bath Temperature on the Contractile Responses of the Large Gut in Adult and Neonate Rats

**DOI:** 10.7759/cureus.46446

**Published:** 2023-10-03

**Authors:** Shuchita Singh, Parul Sharma, Devarshi Dixit, Maloy B Mandal

**Affiliations:** 1 Department of Physiology, Institute of Medical Sciences, Banaras Hindu University, Varanasi, IND; 2 Department of Physiology, Mata Gujri Memorial (MGM) Medical College, Kishanganj, IND

**Keywords:** nω-nitro-l-arginine methyl ester, capsazepine, gut physiology, rectum, colon, temperature, neonatal large gut, large gut contractility

## Abstract

Introduction

Earlier reports on the effect of temperature on gut motility concentrated on experiments conducted on the small intestines of adult animals. The effect of temperature on the large intestine, particularly in neonates, warrants further investigation. The current study investigated the effect of a temperature increase and its mechanism in the colon and rectum of neonate and adult rats.

Methods and materials

In an organ bath preparation, segments from the colon and rectum were subjected to increasing bath temperatures (37°C-40°C). In other groups, pretreatment with capsazepine (1 µM) and Nω-nitro-l-arginine methyl ester (L-NAME) (100 µM) was done, in different groups, to assess their impact on temperature-induced contractile response.

Results

Increasing the bath temperature significantly reduced the contractile tension in the colon and rectum. When L-NAME (100 µM)-pretreated segments of the colon and rectum were subjected to different bath temperatures, the contractile tension increased compared to the contractile tension at different bath temperatures without any drug. Capsazepine (1 µM) pretreatment, on the other hand, enhanced the decrease in the contractile tension in the colon and rectum of adult rats compared to the contractile tension produced at different bath temperatures without any drug, while in neonates, capsazepine (1 µM) pretreatment caused a rise in the contractile tension in the rectum with no effect in the colon. Increased bath temperature from 37°C to 40°C increased the contractile frequency in the colon and rectum in both adult and neonate rats. Pretreatment with L-NAME (100 µM) and capsazepine (1 µM) in adults and L-NAME (100 µM) in neonates caused an increase in the contractile frequency in both the colon and rectum; on the other hand, capsazepine pretreatment did not affect the contractile frequency in the colon and rectum of neonate rats compared to the contractile frequency produced at different bath temperatures without any drug.

Conclusion

The contractile response of rats' large intestines, colon, and rectum to increasing temperatures may involve nitric oxide (NO)-mediated and transient receptor potential vanilloid-1 (TRPV1)-mediated mechanisms. The effects of capsazepine on the colon and rectum of adults and neonates differ, possibly due to the TRPV1-mediated mechanism not developing properly in the neonate and developing later in adulthood.

## Introduction

Body temperature is a critical physiological variable that modifies the functions of several organ systems, including the gastrointestinal system. The temperature may affect mammalian intestinal motility. For a while now, it has been established that higher temperatures lead to more frequent contractions [1,2].

In an earlier study, the temperature sensitivity of rat neonates' large intestines was different from that of adults [3]. In this study, lowering bath temperature from 37°C to 15°C caused temperature-dependent changes in the contractility of the colon and rectum in adult and neonate rats. Also, the neonate rectum seemed more responsive to this temperature change than the colon. In contrast, the adult rectum responded to lowering bath temperature like the adult colon, indicating blunting of the sensitivity of the rectum during the developmental process. Also, this study showed a differential response mechanism to lowering bath temperature in adult and neonate rectum. Some other studies have also explored the impact of low temperatures on gut contractility [4]. Contractile response to a temperature less than the normal body temperature might involve transient receptor potential A1 (TRPA1). TRPA1 is a member of the family of transient receptor potential channels. TRPA1 is known to be activated by noxious cold stimuli [4-6].

Further, high temperature is an important environmental factor affecting gut physiology. The gut may be exposed to high temperatures while consuming hot drinks and food, in conditions of altered physiology such as fever, during exposure to hot climate, etc.

The available literature on how increasing temperatures affect gut motility mainly focuses on experiments on adult animals' small intestines. A study performed on the small intestines of rabbits reported that increasing bath temperature between 22°C and 43°C increased the rhythm of contraction [7]. According to a study conducted on mongrel bitches, high temperatures (45°C) caused a rise in the frequency of the slow wave in the duodenum [8]. In another study, a temperature-dependent increase in contractile frequency but not in amplitude was observed in the small intestines of mice [9]. Many other studies have also reported that the temperature of the drinks and meals affected gastrointestinal motility [10,11].

The impact of temperature on the large gut of newborns, especially on their colon and rectum, remains uncertain.

We hypothesized that neonate large gut sensitivity to high temperatures might differ from adult, and there could be a differential mechanism in the large gut of adult and neonate rats that determines the response of the large gut to increasing bath temperatures, as was observed earlier with lowering bath temperature experiments [3]. In this background, we planned to expose the colon and rectum of adult and neonate rats to a range of temperatures slightly higher than the average body temperature to explore the high temperature-dependent changes in contractility. This study shall help understand the changes that develop in the large gut during development and has implications for understanding the gut motility disorders of neonates.

The present study, therefore, aimed to examine the effects of rising bath temperature on the contractility, in vitro, of the colon and rectum in neonate and adult rats and to discover the likely mechanisms behind it. The following objectives of the study may be defined: (1) to observe the cumulative temperature dose-response (37°C-40°C) on the contractile activity of the colon and rectum in adult and neonate rats and (2) to investigate how increasing bath temperature (37°C-40°C) affects colon and rectum contractile activity in adult and neonate rats.

This study is perhaps the first to record temperature-induced change in the colon and rectum of adult and neonate rats.

## Materials and methods

The present study utilized adult male albino rats aged between four and six months and neonate male rats aged between 10 and 16 days old. The rats belonged to the Charles Foster strain. The room where the animals were kept had its temperature, humidity, and light cycle carefully regulated. A fixed pattern was followed in the light cycle, comprising equal hours of light and darkness. The animals were able to access food and water whenever they needed it. The experiments on animals were carried out in a manner that was completely compliant with the suggestions provided by the Ethical Clearance Committee of the institute (number Dean/1213/CAEC/32). Decapitation was used to sacrifice newborn rats, while adult rats were killed by cervical dislocation. After the animals were sacrificed, a vertical incision was made in the abdomen, and a section of a large gut was dissected. The dissected part of the gut, including the colon and rectum, was thoroughly cleansed, and the contents were flushed out. Following this, a segment of the colon and rectum was placed in a petri dish containing a cold and oxygenated (100%) Krebs-Ringer solution. Longitudinal strips of the colon and rectum measuring 12-15 mm were prepared from these segments and were moved to an organ bath containing 12 mL of continuously oxygenated Krebs-Ringer solution.

During the standardization time, the organ bath's temperature was kept consistent (37°C). The colon and rectum muscle strips were positioned vertically in the organ bath. One end of the muscle strip was fixed to a glass tube and the other to a force transducer (MLT 0210, ADInstruments, Sydney, Australia). After the muscle strip was fixed, a 0.25-0.5 g tension was applied [12,13].

Recording

The mounted preparations were left to equilibrate for 30 minutes (stabilization period), following which control (baseline) data were recorded for another 30 minutes. The contractions recorded were of an isometric type. A bridge amplifier enhanced the contractions. We used an analog interface called PowerLab/4ST system (ADInstruments, Sydney, Australia) to digitize and obtain the data on a computer. The recordings were analyzed using the Chart 5 software designed for Windows by ADInstruments based in Sydney, Australia. The tension (0-10 g) was calibrated before and after the recordings of the contractile responses [12,13].

After recording the control data, the colon and rectum strips were subjected to varying bath temperatures (37°C, 38°C, 39°C, and 40°C) in increasing order, in a cumulative manner, to obtain a temperature dose-response. In other groups, pretreatment with capsazepine (1 µM) or Nω-nitro-l-arginine methyl ester (L-NAME) (100 µM) was done (in different groups), following which the temperature dose-response was observed to understand the impact of the pretreatments on temperature-induced contractile responses [12,13].

Once the recordings were complete, the strip mounted on the organ bath was removed. A blotting paper was used to remove excess water. The damaged parts of the strips were removed by cutting both ends. The weight of the wet tissue was recorded. The contractile tension (gram) was calculated per unit weight (gram) of tissue (gram/gram wet tissue).

Chemicals

The Krebs-Ringer solution was prepared by combining the following ingredients in the indicated amounts (in mM/L): sodium chloride (NaCl) (119), potassium chloride (KCl) (4.7), calcium chloride dehydrate (CaCl_2_.2H_2_O) (2.5), potassium dihydrogen phosphate (KH_2_PO_4_) (1.2), magnesium sulfate heptahydrate (MgSO_4_.7H_2_O) (1.2), sodium carbonate (NaHCO_3_) (5), and glucose (11). The salts used to prepare the Krebs-Ringer solution were of analytical grade and obtained from HiMedia Laboratories Private Limited (Mumbai, Maharashtra, India).

The solution's pH was 7.4. A stock solution (1 mM) of capsazepine was prepared in 100% alcohol, and a stock solution (10 mM) of L-NAME hydrochloride was prepared in distilled water. Later, dilutions of these solutions were made in the Krebs-Ringer solution.

Grouping of animals and experimental protocol

A total of 18 adult and 18 neonate male rats were recruited in the study. The adult rats were arbitrarily divided into Group I, with six rats, and Group II, with 12 rats. Similarly, neonate rats were also arbitrarily divided into Group I, with six rats, and Group II, with 12 rats. In both groups, one rectal and one colon muscle strips were prepared from each rat; n is the number of muscle strips in a set of experiment.

In Group I, in the beginning, a control recording of spontaneous contractions was obtained, following which the muscle strips were subjected to increasing bath temperatures (37°C, 38°C, 39°C, and 40°C) cumulatively, keeping the exposure time 10 minutes for each temperature to obtain the temperature dose-response [3,12,13].

In Group II, six of the 12 rats were randomly selected, and the muscle strips of the colon and rectum were prepared. After recording the spontaneous contractions, the muscle strips were pretreated with capsazepine (1 µM) [13]. Similarly, muscle strips of the colon and rectum prepared from the other six rats were subjected to pretreatment with L-NAME (100 µM) [13]. The exposure time for both pretreatments was 10 minutes, following which the muscle strips were subjected to varying bath temperatures, as mentioned for Group I.

The same protocol was applied to the neonate colon and rectum in Groups I and II.

Statistical analysis

For statistical analysis, the changes in the contractile tension (gram/gram wet tissue) and the contractile frequency (contractions/minute) were expressed as a percentage of the initial (control). The contractile tension at 37°C (control) was considered 100%. Similarly, the contractile frequency at 37°C was considered 100%.

The GraphPad Prism 4 software (GraphPad Software, San Diego, CA) was used for statistical analysis. The groups were compared using a two-way analysis of variance (ANOVA) to determine dose-response relationships. Also, Student's t-test was used as required, and a p value of less than 0.05 was considered significant.

## Results

The effect of increasing bath temperature (37°C-40°C) on the contractile tension of the colon and rectum

**Figure 1:**
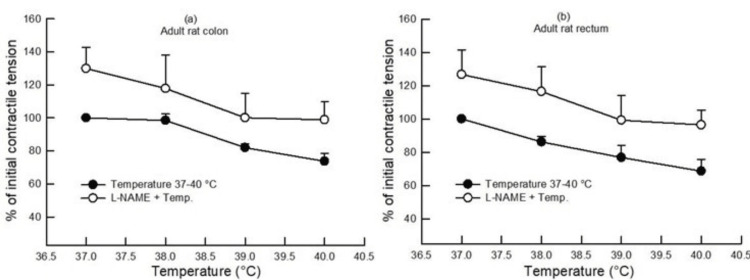
Dose-response curve showing the effect of increasing bath temperature (37°C, 38°C, 39°C, and 40°C) on the contractile tension (percentage of initial) in the colon (a) and rectum (b) of adult rats with and without pretreatment with L-NAME (100 µM). An increase in bath temperature decreased the contractile tension as compared to 37°C (p<0.05, Student's t-test, paired, n=6), and L-NAME pretreatment caused an increase in the contractile tension in both the colon and rectum (p<0.05, two-way ANOVA, n=4). Data points indicate mean±SEM values. L-NAME, Nω-nitro-l-arginine methyl ester; ANOVA, analysis of variance; SEM, standard error of the mean; Temp, temperature

In the adult large gut samples, a rise in bath temperature from 37°C to 40°C caused a decrease in the contractile tension (gram/gram wet tissue) in the colon and rectum by 26% and 31%, respectively, as compared to the contractile tension produced at 37°C, i.e., 100% (p<0.05, Student's t-test, paired, n=6) (Figure 1). When compared mutually, the effect of increasing bath temperature on the contractile tension was similar (p>0.05, two-way ANOVA, n=6) in the colon and rectum.

**Figure 2:**
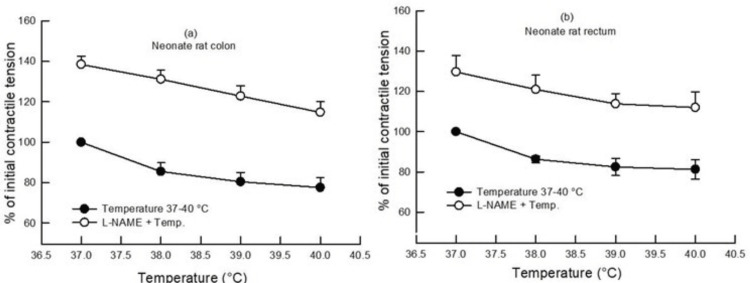
Dose-response curve showing the effect of rising bath temperature (37°C, 38°C, 39°C, and 40°C) on the contractile tension (percentage of initial) in the colon (a) and rectum (b) of neonate rats with and without pretreatment with L-NAME (100 µM). An increase in bath temperature decreased the contractile tension as compared to 37°C (p<0.05, Student's t-test, paired, n=6). L-NAME pretreatment caused an increase in the contractile tension in both the colon and rectum (p<0.05, two-way ANOVA, n=7). Data points indicate mean±SEM values. L-NAME, Nω-nitro-l-arginine methyl ester; ANOVA, analysis of variance; SEM, standard error of the mean; Temp, temperature

In the neonate large gut samples, an increase in bath temperature from 37°C to 40°C also reduced the contractile tension (gram/gram wet tissue) in the colon and rectum by 22% and 19% as compared to the contractile tension produced at 37°C, i.e., 100% (p<0.05, Student's t-test, paired, n=6) (Figure 2). When the effect of increasing bath temperature on the contractile tension of the colon and rectum was compared, it was found to be similar (p>0.05, two-way ANOVA, n=6).

The effect of increasing bath temperature (37°C-40°C) on the contractile tension after pretreatment with L-NAME (100 µM) and capsazepine (1 µM) in the colon and rectum

**Figure 3:**
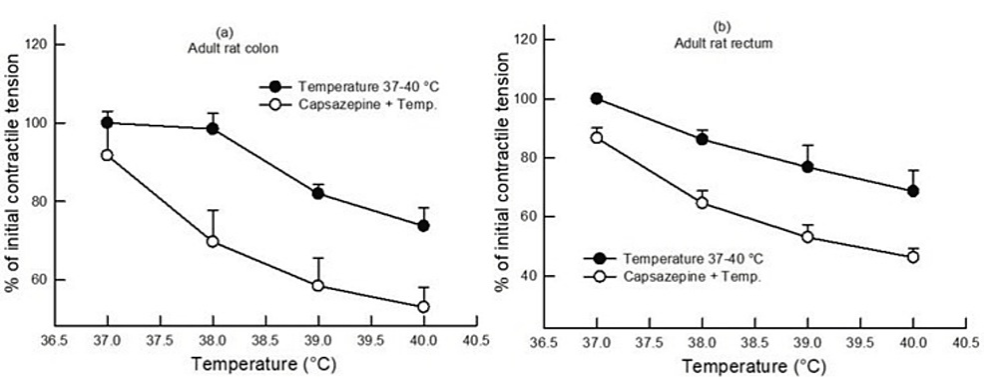
Dose-response curve showing the effect of change in bath temperature (37°C, 38°C, 39°C, and 40°C) on the contractile tension (percentage of initial) in the colon (a) and rectum (b) of adult rats with and without pretreatment with capsazepine (1 µM). Capsazepine pretreatment produced a decrease in the contractile tension in the colon and rectum of adult rats (p<0.05, two-way ANOVA, n=4). Data points indicate mean±SEM values. ANOVA, analysis of variance; SEM, standard error of the mean; Temp, temperature

When segments of the colon and rectum of adult rats were subjected to different bath temperatures after pretreatment with L-NAME (100 µM), it was observed that there was a significant increase in the contractile tension as compared to the contractile tension (gram/gram wet tissue) at different bath temperatures in the absence of any drug (p<0.05, two-way ANOVA, n=4-6) (Figure 1), indicating that L-NAME pretreatment reduced the relaxing effect produced by the increase in bath temperature. On the other hand, capsazepine (1 µM) pretreatment caused a decrease in the contractile tension (gram/gram wet tissue) as compared to the contractile tension (gram/gram wet tissue) produced at different bath temperatures in the absence of any drug (p<0.05, two-way ANOVA, n=4-6) (Figure 3), indicating that capsazepine pretreatment enhanced the relaxing effect produced by the increase in bath temperature.

**Figure 4:**
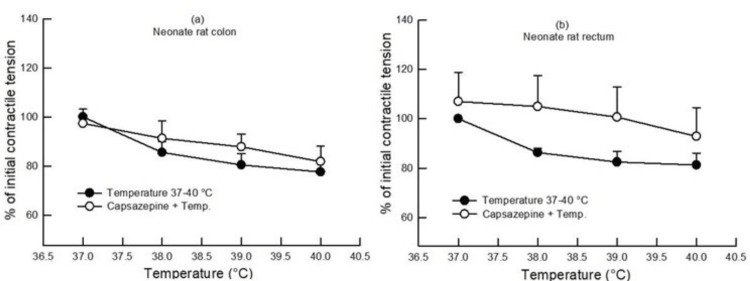
Dose-response curve showing the effect of change in bath temperature (37°C, 38°C, 39°C, and 40°C) on the contractile tension (percentage of initial) in the colon (a) and rectum (b) of neonate rats after pretreatment with capsazepine (1 µM). The contractile tension increased only in the neonate rectum after capsazepine pretreatment (p<0.05, two-way ANOVA, n=6). Data points indicate mean±SEM values. ANOVA, analysis of variance; SEM, standard error of the mean; Temp, temperature

In neonates, L-NAME (100 µM) pretreatment in the colon and rectum also increased the contractile tension (gram/gram wet tissue) as compared to the contractile tension (gram/gram wet tissue) at different bath temperatures in the absence of any drug (p<0.05, two-way ANOVA, n=7) (Figure 2), whereas capsazepine (1 µM) pretreatment caused an increase in the contractile tension (gram/gram wet tissue) in the neonate rectum (Figure 4) and no change in the colon.

The effect of increasing bath temperature (37°C-40°C) on the contractile frequency of the colon and rectum

**Figure 5:**
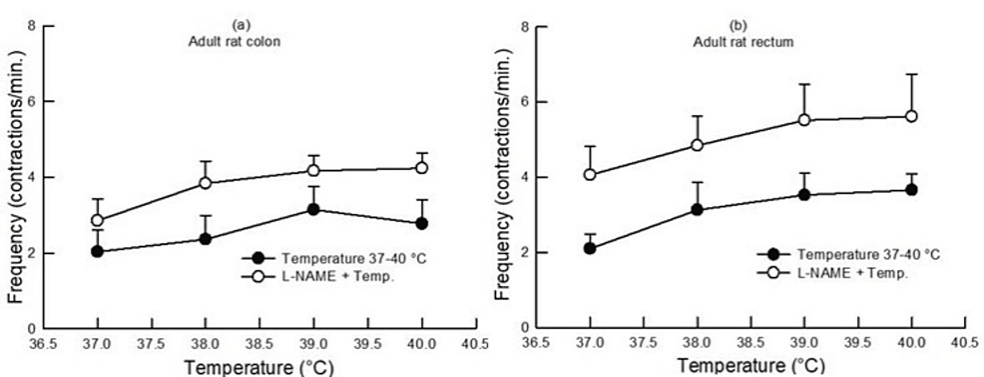
Dose-response curve showing the effect of change in bath temperature (37°C, 38°C, 39°C, and 40°C) on the contractile frequency in the colon (a) and rectum (b) of adult rats with and without pretreatment with L-NAME (100 µM). An increase in bath temperature caused an increase in the contractile frequency. Pretreatment with L-NAME increased the contractile frequency in both the colon and rectum of adult rats (p<0.05, two-way ANOVA, n=6-7). Data points indicate mean±SEM. L-NAME, Nω-nitro-l-arginine methyl ester; ANOVA, analysis of variance; SEM, standard error of the mean; Temp, temperature

In the colon and rectum of adult rats, there was an increase in contractions per minute compared to 37°C, as bath temperature was raised from 37°C to 40°C. Further, the effect of increasing bath temperature was similar (p>0.05, two-way ANOVA, n=7) (Figure 5).

**Figure 6:**
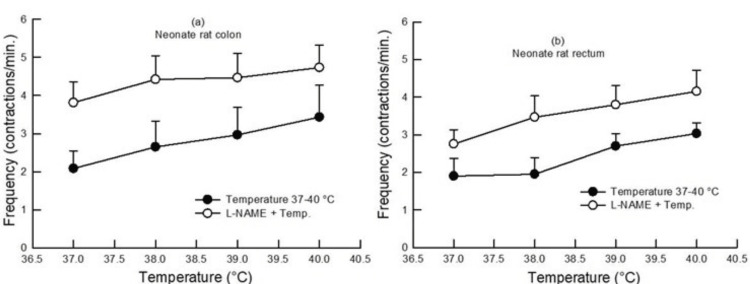
Dose-response curve showing the effect of increasing bath temperature (37°C, 38°C, 39°C, and 40°C) on the contractile frequency in the colon (a) and rectum (b) of neonate rats with and without pretreatment with L-NAME (100 µM). An increase in bath temperature caused an increase in the contractile frequency. Pretreatment with L-NAME caused an increase in the contractile frequency in the colon and rectum (p<0.05, two-way ANOVA, n=6-9). Data points indicate mean±SEM values. L-NAME, Nω-nitro-l-arginine methyl ester; ANOVA, analysis of variance; SEM, standard error of the mean; Temp, temperature

In neonate rats, increased bath temperature from 37°C to 40°C enhanced the contractile frequency in the large gut, colon, and rectum segments. As in adults, the effect of increasing bath temperature was similar in the colon and rectum (p>0.05, two-way ANOVA, n=6-9) (Figure 6).

The effect of increasing bath temperature (37°C-40°C) on the contractile frequency after pretreatment with L-NAME (100 µM) and capsazepine (1 µM) in the colon and rectum

**Figure 7:**
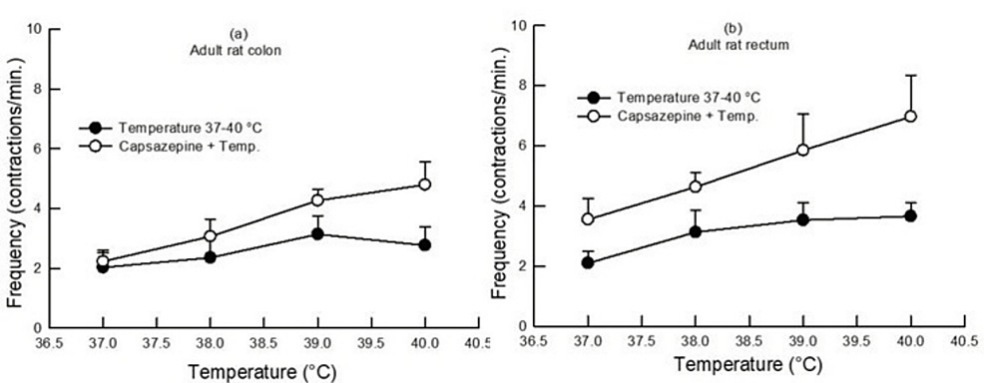
Dose-response curve showing the effect of increasing bath temperature (37°C, 38°C, 39°C, and 40°C) on the contractile frequency in the colon (a) and rectum (b) of adult rats with and without pretreatment with capsazepine (1 µM). Capsazepine pretreatment caused an increase in the contractile frequency in the colon and rectum of adult rats (p<0.05, two-way ANOVA, n=6). Data points indicate mean±SEM values. ANOVA, analysis of variance; SEM, standard error of the mean; Temp, temperature

In adult rats, both L-NAME (100 µM) and capsazepine (1 µM) pretreatment increased the contractile frequency in the colon and rectum, as compared to the contractile frequency produced at different bath temperatures in the absence of any drug (p<0.05, two-way ANOVA, n=6-7) (Figures 5, 7).

**Figure 8:**
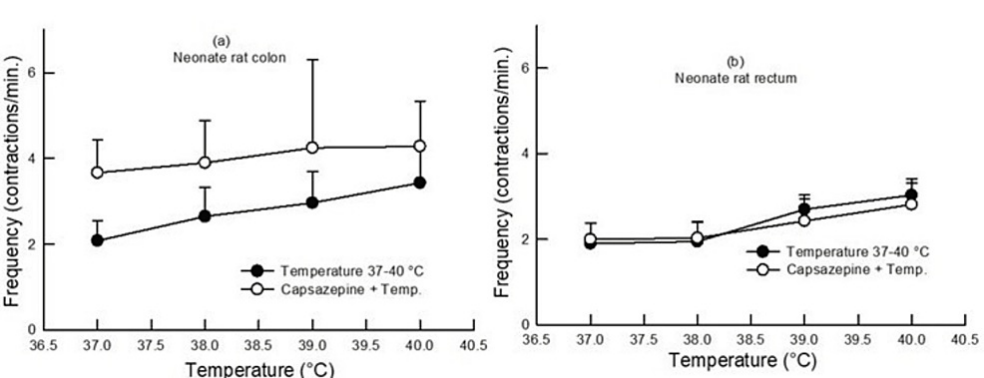
Dose-response curve showing the effect of change in bath temperature (37°C, 38°C, 39°C, and 40°C) on the contractile frequency in the colon (a) and rectum (b) of neonate rats before and after pretreatment with capsazepine (1 µM). Capsazepine pretreatment did not produce any change in the contractile frequency in the colon and rectum of neonate rats (p>0.05, two-way ANOVA, n=6). Data points indicate mean±SEM values. ANOVA, analysis of variance; SEM, standard error of the mean; Temp, temperature

In neonate rats, pretreatment with L-NAME (100 µM) increased the contractile frequency in the colon and rectum as compared to the contractile frequency produced in the absence of any drug at different bath temperatures (p<0.05, two-way ANOVA, n=6-9) (Figure 6). At the same time, capsazepine pretreatment did not produce any change in the contractile frequency in the colon and rectum as compared to the contractile frequency produced in the absence of a drug at different bath temperatures (p>0.05, two-way ANOVA, n=6) (Figure 8).

## Discussion

This study examined the influence of rising bath temperature on the in vitro contractility of the colon and rectum in rats. A rise in bath temperature from 37°C to 40°C caused a progressive decline in the contractile tension of the colon and rectum in both adult and neonate rats (Figures 1, 2). Also, the colon and rectum responded similarly to increasing bath temperature in adults and neonates.

An increase in the contractile frequency of the colon and rectum was observed with rising bath temperature in both adult and neonate rats, without any difference between the colon and rectum (Figures 5, 6).

A few earlier reports state the effect of temperature change on motility in different parts of the gut. One study on healthy young males observed that drinking 500 mL of water at 2°C one hour before a meal more effectively reduced gastric contractions than drinking the same amount at 37°C or 60°C. The gastric contractile frequency after one hour of drinking water was less in the 2°C test than in the 60°C test [14]. In another study, drinking a warm (60°C) carbohydrate-protein drink post exercise increased gastric emptying, unlike a cold (4°C) beverage, in healthy young males. The drink's temperature appears important in gastric-emptying postexercise rate [15]. A study on young rats showed that gastric emptying and intestinal transit were significantly enhanced in heat-exposed animals [16]. In an investigation, esophageal motility was influenced by the temperature of the water bolus [17]. According to one report, irrigation with warm water during colonoscopy suppressed pain significantly [18]. Recently, an association was reported between the cool-temperature-dependent attenuation of peristalsis in the colon and the activation of transient receptor potential melastatin 8 [19].

It may be reiterated that this present investigation is perhaps the first to record the high-temperature-induced change in the colon and rectum of adults and neonates.

To understand the mechanism of temperature-induced relaxation, we used L-NAME, an inhibitor of nitric oxide synthase (NOS), and capsazepine, a transient receptor potential vanilloid-1 (TPRV1) receptor inhibitor.

Pretreatment with L-NAME inhibited the relaxing effect produced by increasing bath temperature, as evidenced by the enhancement of the contractile tension in both the colon and rectum of adult rats (Figure 1), indicating the role of nitric oxide (NO) in high-temperature-mediated relaxation of the colon and rectum in adult rats. NO has been known to cause the relaxation of the gastrointestinal tract (GIT) smooth muscle [20-24]. The current experiments may have been the first to show that a NO mechanism mediates temperature-induced relaxation.

Capsaicin, a TRPV1 agonist, has an intense influence on thermoregulation, affecting only the warm-sensitive side of the regulatory system [25]. Capsaicin is the main spicy component of red pepper [26]. It acts through the transient receptor potential vanilloid-1 (TRPV1) ion channel [27]. In the digestive system, TRPV1 is distributed mainly in the submucosal and myenteric nerve plexus, mucosa, and mucosal, parietal, and antral G cells of the stomach [27]. TRPV1 may be involved in regulating gut motility and may also be involved in irritable bowel syndrome, gastric ulcer, and functional dyspepsia [27]. In addition to capsaicin, TRPV1 is also activated by mechanical stimulation, ethyl alcohol, mediators of inflammation, tissue damage, low pH, high temperature, changes in extracellular osmotic pressure, an intracellular redox state, substance P, nerve growth factor, and prostaglandins [27]. A TRPV1 antagonist can block activated TRPV1 [28]. Capsazepine is a synthetic capsaicin analog and can act as a potent blocker of TRPV1 channels [18].

In the present study, capsazepine pretreatment potentiated temperature-induced relaxation in adult rats' colon and rectum (Figure 3). Thus, TRPV1 may be involved in temperature-induced contractile activity.

In the case of neonates, when the colon and rectum were preincubated with L-NAME, an augmentation in the contractile tension was seen, similar to its effect in adults (Figures 1, 2). In contrast, the pre-application of capsazepine inhibited high-temperature-induced relaxation in the neonate rectum without impacting the neonate colon (Figure 4). Thus, the effect of the pre-application of capsazepine on the neonate rectum was opposite to its impact on the adult rectum. During development, the change in the contractile response of the colon and rectum to capsazepine might have occurred. It may be speculated that there is a change in the TRPV1 mechanism or receptor distribution during development from neonate to adulthood.

Further, pretreatment with L-NAME and capsazepine increased the contractile frequency in adult rats' colons and rectum (Figures 5, 7). This result indicated that NO and TRPV1 have depressing effects on frequency.

In neonate rat gut samples, there was also an increase in the colon and rectum contractile frequency (pretreatment with L-NAME, 100 µM) in response to increasing bath temperature, as seen in the colon and rectum of adult rats (Figure 6). This action in neonates was similar to that in adults. At the same time, capsazepine pretreatment did not change the contractile frequency in the colon and rectum of neonate rats (Figure 8). This distinct effect of capsazepine on adults and neonates suggests that TRPV1-mediated mechanisms were underdeveloped in neonates and may only fully develop after adulthood.

A few other studies have compared the contractility among adult and neonate rat gut and signified some differences, which could be due to developmental changes. An in vitro study on the rat gut found that the contractile responses caused by acetylcholine were comparable in adults and neonates, but the blocking effect of atropine in the colon was more evident in adults than in neonates. Also, this study showed that the mechanism of action of histamine was not the same in adults and neonates, as a significant increase of contractions by pheniramine was found only in the adult rectum. This study indicates different cholinergic and histaminergic activities in adults and neonates and rectal and colonic tissues [29]. In another study conducted on the rat gut, it was observed that 5-hydroxytryptamine brought the concentration-dependent contractions of the ileum irrespective of age.

On the contrary, these contractions were diminished by pretreatment with atropine only in neonate rats [30]. Lately, we reported [3] that the absence of capsaicin-induced contractile tension in the neonate colon and rectum could be because of capsaicin receptors being late during the postnatal gut development process. It was concluded that capsaicin-induced changes in contractile activity may or may not involve TRPV1 or the NO pathway, depending on the part of the large gut (colon or rectum) and developmental maturity (neonate or adult).

Although the current investigation successfully identified that the involvement of TRPV1 and nitric oxide in temperature brought changes in gut contractions, the exact mechanisms by which the contractile machinery is affected could not be ascertained. Further, the differential involvement of TRPV1 in the adult and neonate rectum in this study indicated the presence of the developmental issue but could not be explained adequately. Extended research using immune histochemical and molecular marker tools to explore the changes in TRPV1 receptors during development may address these issues. This is a limitation of the present investigation. Also, how temperature exposures were carried out might introduce potential confounding effects due to tissue adaptation or cumulative stress over time. Considering this as a possibility, this is also one of the limitations of the present study. Additionally, in the present study, the per se effect of capsazepine and L-NAME on the contraction of the colon and rectum was not studied. We compared how temperature affects large gut contractility to how pretreatment with capsazepine and L-NAME involves temperature-induced changes in the contraction of the colon and rectum.

## Conclusions

Thus, increasing bath temperature reduced the contractile tension and augmented the contractile frequency in the colon and rectum of rats, both adults and neonates. This contractile response to raised temperature may involve NO- and TRPV1-mediated mechanisms. Further, the effects of capsazepine on adults and neonates are different. This may be due to the TRPV1 mechanism not being properly developed in the neonate, and this may develop later in the process of attaining adulthood.

## References

[1] Fintl C, Hudson NP, Handel I, Pearson GT (2016). The effect of temperature changes on in vitro slow wave activity in the equine ileum. Equine Vet J.

[2] Liu JY, Du P, Chan WY, Rudd JA (2019). Use of a microelectrode array to record extracellular pacemaker potentials from the gastrointestinal tracts of the ICR mouse and house musk shrew (Suncus murinus). Cell Calcium.

[3] Singh S, Mandal MB (2020). In vitro study of effect of low temperature treatment on contraction of colon and rectum of adult and neonate rats. Intern J Zool Invest.

[4] Dong Y, Shi HL, Shi JR, Wu DZ (2010). Transient receptor potential A1 is involved in cold-induced contraction in the isolated rat colon smooth muscle. Sheng Li Xue Bao.

[5] Karashima Y, Talavera K, Everaerts W (2009). TRPA1 acts as a cold sensor in vitro and in vivo. Proc Natl Acad Sci U S A.

[6] McKemy DD, Neuhausser WM, Julius D (2002). Identification of a cold receptor reveals a general role for TRP channels in thermosensation. Nature.

[7] Chang PY, Hsu FY (1942). The chemical excitability of the isolated rabbit small intestine. Q J Exp Physiol Cogn Med Sci.

[8] Milton GW, Smith AW (1956). The pacemaking area of the duodenum. J Physiol.

[9] Studier EH, Behrend TA, Freed AL (1976). Effect of temperature on intrinsic intestinal motility in a hibernator. J Thermal Biol.

[10] Sun WM, Houghton LA, Read NW, Grundy DG, Johnson AG (1988). Effect of meal temperature on gastric emptying of liquids in man. Gut.

[11] Sun WM, Penagini R, Hebbard G (1995). Effect of drink temperature on antropyloroduodenal motility and gastric electrical activity in humans. Gut.

[12] Kumari Nirja, Sharma P, Tiwari AK, Mandal MB (2018). Plastic toxin bisphenol-a depresses the contractile activity of rat ileum and colon in vitro. Indian J Physiol Pharmacol.

[13] Singh S, Sharma P, Dixit D, Mandal MB (2023). Capsaicin fails to produce changes in contractile tension in large gut of neonate rats. Indian J Physiol Pharmacol.

[14] Fujihira K, Hamada Y, Yanaoka T, Yamamoto R, Suzuki K, Miyashita M (2020). The effects of water temperature on gastric motility and energy intake in healthy young men. Eur J Nutr.

[15] Fujihira K, Takahashi M, Shimamura K, Hayashi N (2022). Effects of different temperatures of carbohydrate-protein-containing drinks on gastric emptying rate after exercise in healthy young men: randomized crossover trial. J Physiol Anthropol.

[16] Datta UK (2001). Effect of heat stress on gastro-intestinal motility in young albino rats. Indian J Physiol Pharmacol.

[17] Choi YJ, Park MI, Park SJ (2014). The effect of water bolus temperature on esophageal motor function as measured by high-resolution manometry. Neurogastroenterol Motil.

[18] Church JM (2002). Warm water irrigation for dealing with spasm during colonoscopy: simple, inexpensive, and effective. Gastrointest Endosc.

[19] Sugino S, Inoue K, Kobayashi R (2022). Association between the cool temperature-dependent suppression of colonic peristalsis and transient receptor potential melastatin 8 activation in both a randomized clinical trial and an animal model. J Neurogastroenterol Motil.

[20] Guerra DD, Bok R, Vyas V, Orlicky DJ, Lorca RA, Hurt KJ (2019). Akt phosphorylation of neuronal nitric oxide synthase regulates gastrointestinal motility in mouse ileum. Proc Natl Acad Sci U S A.

[21] Idrizaj E, Traini C, Vannucchi MG, Baccari MC (2021). Nitric oxide: from gastric motility to gastric dysmotility. Int J Mol Sci.

[22] Parsons SP, Huizinga JD (2020). Nitric oxide is essential for generating the minute rhythm contraction pattern in the small intestine, likely via ICC-DMP. Front Neurosci.

[23] Groneberg D, Voussen B, Friebe A (2016). Integrative control of gastrointestinal motility by nitric oxide. Curr Med Chem.

[24] Temiz TK, Demir O, Simsek F (2016). Effect of nitrergic system on colonic motility in a rat model of irritable bowel syndrome. Indian J Pharmacol.

[25] Szolcsányi J (2015). Effect of capsaicin on thermoregulation: an update with new aspects. Temperature (Austin).

[26] Liu T, Wan Y, Meng Y, Zhou Q, Li B, Chen Y, Wang L (2023). Capsaicin: a novel approach to the treatment of functional dyspepsia. Mol Nutr Food Res.

[27] Du Q, Liao Q, Chen C, Yang X, Xie R, Xu J (2019). The role of transient receptor potential vanilloid 1 in common diseases of the digestive tract and the cardiovascular and respiratory system. Front Physiol.

[28] Yang MH, Jung SH, Sethi G, Ahn KS (2019). Pleiotropic pharmacological actions of capsazepine, a synthetic analogue of capsaicin, against various cancers and inflammatory diseases. Molecules.

[29] Singh S, Mandal MB (2013). In vitro study of acetylcholine and histamine induced contractions in colon and rectum of adult and neonate rats. Indian J Physiol Pharmacol.

[30] Lobo SB, Denyer M, Britland S, Javid FA (2011). The involvement of the serotonergic transmission system in neonatal and adult rat ileum contractility varies with age. Pharmacology.

